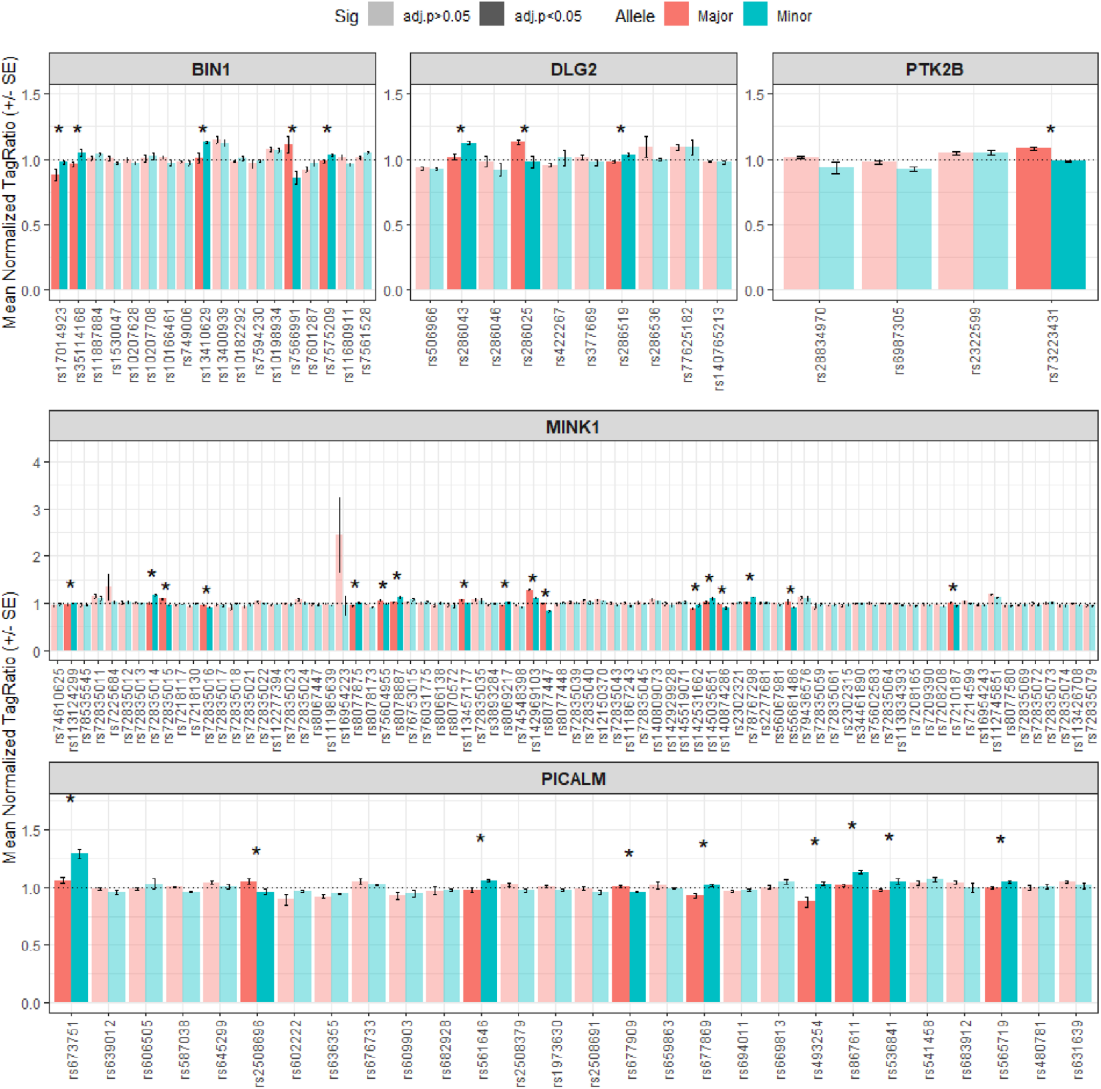# Evaluation of the functional activity in synaptic genes related to polygenic risk for Alzheimer’s disease using a Massively Parallel Reporter Assay

**DOI:** 10.1002/alz.090718

**Published:** 2025-01-03

**Authors:** Danna Perlaza, Laia Lidón Gil, Alba Cervantes‐González, Sara Serrano‐Requena, Natalia Valle Tamayo, Érika Sánchez‐Aced, Esther Álvarez‐Sánchez, Joaquim Aumatell Escabias, Laia Muñoz, Sonia Sirisi Dolcet, Oriol Dols‐Icardo, Alberto Lleo, Olivia Belbin

**Affiliations:** ^1^ Sant Pau Memory Unit, Hospital de la Santa Creu i Sant Pau ‐ Biomedical Research Institute Sant Pau ‐ Universitat Autònoma de Barcelona, Barcelona Spain; ^2^ Center for Biomedical Investigation Network for Neurodegenerative Diseases (CIBERNED), Madrid Spain; ^3^ Memory Unit, Department of Neurology, Institut de Recerca Sant Pau ‐ Hospital de Sant Pau, Universitat Autònoma de Barcelona, Barcelona, Spain. Centro de Investigación Biomédica en Red en Enfermedades Neurodegenerativas (CIBERNED), Madrid, Spain, Barcelona, Barcelona Spain

## Abstract

**Background:**

Synaptic degeneration is a primary neuropathological factor associated with cognitive decline in Alzheimer’s disease (AD). In 2021, we generated a synaptic Polygenic Risk Score (PRS) that comprised only 8 variants within 6 synaptic genes (*APOE*, *PICALM*, *BIN1*, *PTK2B*, *DLG2* and *MINK1*) that predicted AD with 72% accuracy in two neuropathological cohorts. This supports the hypothesis that genetic variants that regulate an individual’s vulnerability to AD‐related synapse degeneration could be used to identify individuals at‐risk for AD prior to the appearance of clinical symptoms. The *aim* of this study was to determine whether the constituent variants of the linkage disequilibrium (LD) blocks represented by the PRS have regulatory activity *in vitro*.

**Method:**

We cloned an oligonucleotide library of 137 putative regulatory variants each represented by 5 barcodes per allele into pMPRA1 vector. Then we transfected the plasmids into HEK293 cells (n = 5). We extracted DNA and RNA from the cells and sequenced on an Illumina MiSeq. Using the *mpra* package in R, we normalized the tag counts to a common size of 10 million reads and computed paired log ratios of RNA/DNA counts for each barcode. We used weighted linear models to test for differential activity of the minor versus the major allele of each SNP using the *mpralm* function, adjusting for multiple testing using the Benjamini‐Hochberg method.

**Result:**

We acquired approximately 15 million reads of DNA and RNA from 5 independent experiments. We found that 35 out of the 137 SNPs tested had differential activity between alleles (adj. p <0.05, Figure 1A). Three of the SNPs that showed regulatory activity were included in the PRS (*BIN1*: rs17014923 and rs35114168; *DLG2*: rs286043) and the remaining 32 are captured on LD blocks within the synaptic PRS.

**Conclusion:**

All LD captured by the synaptic PRS contain SNPs that impact on regulatory activity, thus supporting a potential mechanism by which changes in the expression of these specific loci that encode synaptic proteins could lead to a modified cumulative risk for AD. Further studies to determine the precise mechanism involved in this regulatory activity at the synapse could guide future therapeutic strategies for AD.